# In Vivo Two-Photon Imaging of Axonal Dieback, Blood Flow, and Calcium Influx with Methylprednisolone Therapy after Spinal Cord Injury

**DOI:** 10.1038/srep09691

**Published:** 2015-05-19

**Authors:** Peifu Tang, Yiling Zhang, Chao Chen, Xinran Ji, Furong Ju, Xingyu Liu, Wen-Biao Gan, Zhigang He, Shengxiang Zhang, Wei Li, Lihai Zhang

**Affiliations:** 1 Department of Orthopedics, the General Hospital of Chinese People's Liberation Army, Beijing, China, 100853; 2 Key Laboratory of Chemical Genomics, Shenzhen Graduate School, Peking University, Shenzhen, China, 518055; 3 Skirball Institute, Department of Neuroscience and Physiology, New York University School of Medicine, New York, USA, 10016; 4 School of Life Sciences, Lanzhou University, Lanzhou, China, 73000; 5 Beijing YouAn Hospital, Capital Medical University, Beijing, China, 100069; 6 F.M. Kirby Program in Neuroscience, Children's Hospital Boston, Harvard Medical School, Boston, Massachusetts, USA, 02115

## Abstract

Severe spinal cord injury (SCI) can cause neurological dysfunction and paralysis. However, the early dynamic changes of neurons and their surrounding environment after SCI are poorly understood. Although methylprednisolone (MP) is currently the standard therapeutic agent for treating SCI, its efficacy remains controversial. The purpose of this project was to investigate the early dynamic changes and MP's efficacy on axonal damage, blood flow, and calcium influx into axons in a mouse SCI model. YFP H-line and Thy1-GCaMP transgenic mice were used in this study. Two-photon microscopy was used for imaging of axonal dieback, blood flow, and calcium influx post-injury. We found that MP treatment attenuated progressive damage of axons, increased blood flow, and reduced calcium influx post-injury. Furthermore, microglia/macrophages accumulated in the lesion site after SCI and expressed the proinflammatory mediators iNOS, MCP-1 and IL-1β. MP treatment markedly inhibited the accumulation of microglia/macrophages and reduced the expression of the proinflammatory mediators. MP treatment also improved the recovery of behavioral function post-injury. These findings suggest that MP exerts a neuroprotective effect on SCI treatment by attenuating progressive damage of axons, increasing blood flow, reducing calcium influx, and inhibiting the accumulation of microglia/macrophages after SCI.

Spinal cord injury (SCI) is a devastating medical problem that causes serious disability and paralysis. Approximately 40 million people worldwide experience SCI every year^1^. The primary injury is caused by traumatic spinal cord damage^2^. The secondary injury can destroy nearby neurons that were not damaged during the primary injury^3^. After the initial damage of the blood vessels in a spinal cord region, secondary injury causes a fall in microvascular blood flow that leads to ischemia and hypoxia, which exacerbate the primary injury^4^. In previous studies, spinal cord blood flow was often measured by Doppler ultrasound^5^. However, Doppler ultrasound can only measure blood vessels of approximately 100 μm in diameter^6^, damage to regional microvascular blood flow proximal to lesion site remains poorly understood. In addition, an increase in intracellular free [Ca^2+^] results in the activation of the calcium-activated protease calpain, which is involved in neuronal apoptosis^7^. However, the changes of calcium influx in injured axons of living animal after SCI remains unclear. Furthermore, the role of microglia in SCI has been controversial with both beneficial and destructive effects^8^. Microglia can phagocytose cellular debris after SCI. They also can infiltrate and accumulate at the injured epicenter and secrete proinflammatory cytokines, which may aggravate secondary SCI^9^.

To reduce secondary injury after SCI, clinical and experimental studies have been conducted to block the development of these abnormalities. Ecto-domain phosphorylation^10^ and fluoxetine treatment^11^ have been reported as potential methods for functional recovery after SCI. Although the effects of these therapeutic regimens are compelling, their clinical applications are limited. After the first demonstration of the experimental efficacy of high dose methylprednisolone (MP) in acute experimental SCI^12^, MP has been widely used in clinical treatment for SCI patients^13^. However, recent retrospective cohort studies have demonstrated a lack of statistical difference between SCI patients treated with and without MP^14^. The efficacy of MP in SCI treatment remains controversial.

In previous laboratory studies, axons were assessed by biotinylated dextran amine (BDA) tract tracing^15^, and the intracellular calcium concentration in the injured spinal cord was measured using the techniques of La^3+^ blockage and atomic absorption spectroscopy^16^. For these *in vitro* methods, tissue must be extracted from the spinal cord. For these reasons, the early dynamic changes of SCI and MP's effect governing secondary injury remain unclear. In the present study, we took advantage of two-photon microscopy and spinal cord implanted window, which are able to image axonal dieback in the living mouse spinal cord over multiple hours. We also performed *in vivo* imaging of the regional microvascular blood flow and calcium influx into axons at the edge of lesion site^17^. These *in vivo* methods allowed us to further our understanding of early dynamic changes, as well as MP's effect on axonal damage, microvascular blood flow, and calcium influx into axons after SCI.

## Results

### MP attenuated axonal damage and neuronal death

**Figure 1:**
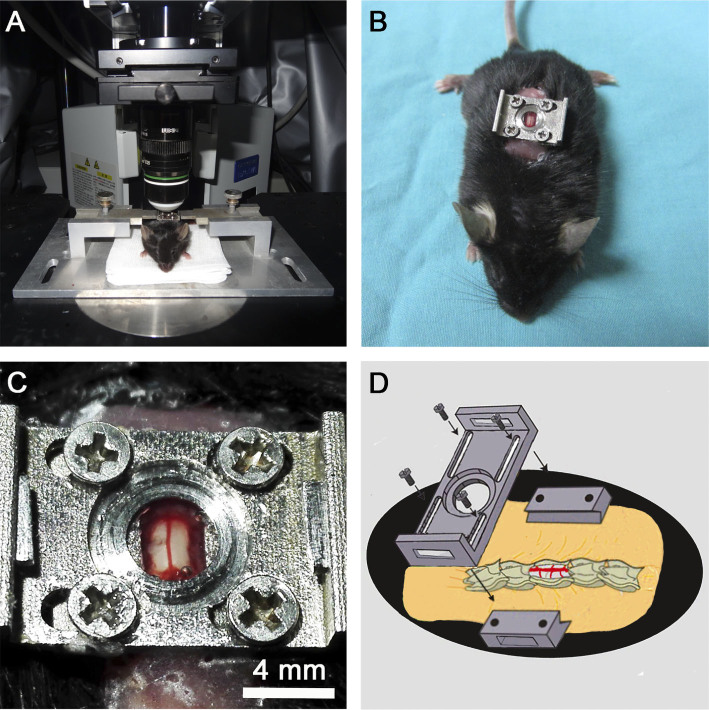
In vivo two-photon imaging of the mouse spinal cord. (A) The customized spinal stabilization device with an implanted window. (B) The mouse with an implanted window. (C) The segment of the spinal cord exposed for two-photon imaging. (D) The schema showing the implanted window on the exposed T12 spinal cord segment.

**Figure 2:**
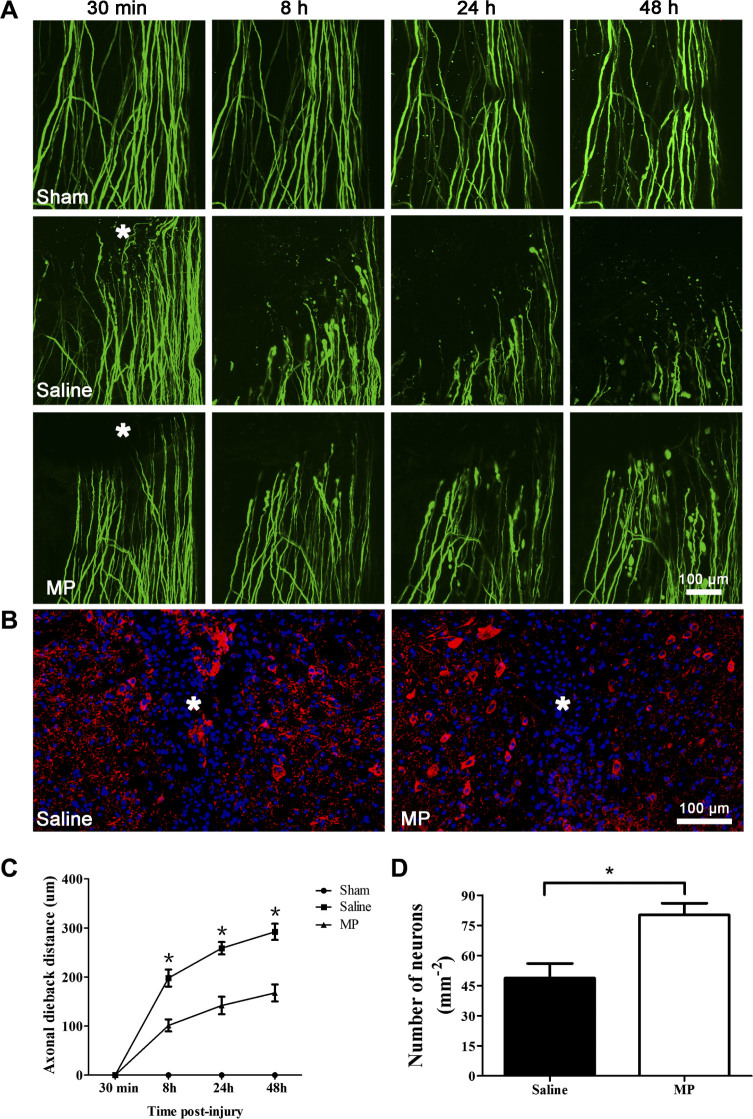
MP attenuated axonal damage and neuronal death after SCI. (A) In vivo two-photon imaging of axonal dieback after hemisection spinal cord injury. Representative images of axons in the sham group (n = 6) at 30 min, 8 h, 24 h, and 48 h post-surgery. Representative images of axonal dieback in the saline-treated SCI group and the MP-treated SCI group at 30 min, 8 h, 24 h, and 48 h post-injury. Asterisk indicates lesion site. (B) Representative MAP-2 (red) and DAPI (blue) staining reveals the effects of MP on neurons in the saline-treated group and the MP-treated group. Asterisk indicates lesion site. (C) The axonal dieback distance from initial lesion site after hemisection SCI in the saline-treated SCI group (n = 6), the MP-treated SCI group (n = 6) and the sham group (n = 6). Fifteen to twenty axons were measured per animal. Values presented are mean ± SEM. *P < 0.01. Repeated measure ANOVA followed by Fisher's LSD. (D) The number of neurons at the edge of lesion site in saline-treated group (n = 6) and MP-treated group (n = 6). Values presented are mean ± SEM. *P < 0.01, P = 0.007. Statistical comparision was done using Student's *t* test.

We used two-photon microscopy to image the axonal dieback in the living mouse spinal cord and investigate the effect of MP treatment after hemisection SCI (Fig. 1). Our results showed that the axons in the sham group (n = 6) remained intact during all imaging sessions after surgery. The severed axons dieback from the initial lesion site over time after hemisection injury (Fig. 2A). We first imaged the injured axons at 30 min post-injury and measured the axonal dieback distance from the initial lesion site. The respective axonal average dieback distances from the initial lesion site at 8 h, 24 h and 48 h were 197.95 ± 42.87 μm, 258.72 ± 30.79 μm, 292.26 ± 40.54 μm in the saline-treated SCI group (n = 6), and 101.29 ± 29.89 μm, 142.04 ± 43.75 μm, 167.58 ± 42.41 μm in the MP-treated SCI group (n = 6), respectively (Fig. 2C). At each time point, the saline-treated group exhibited a greater axonal dieback distance than the MP-treated group (P < 0.01 for all). To investigate the pathological changes and MP's effect on deep tissue after SCI, we measured the number of neurons at the edge of lesion site 3 days post-injury (Fig. 2B). The number of neurons was 48.71 ± 7.26 cells/mm^2^ in the saline-treated group (n = 6) and 80.21 ± 5.76 cells/mm^2^ in the MP-treated group (n = 6). The number of neurons was greater in the MP group than in the saline group (Fig. 2D, P = 0.007).

### MP increased regional microvascular blood flow and reduced microvessel loss

**Figure 3:**
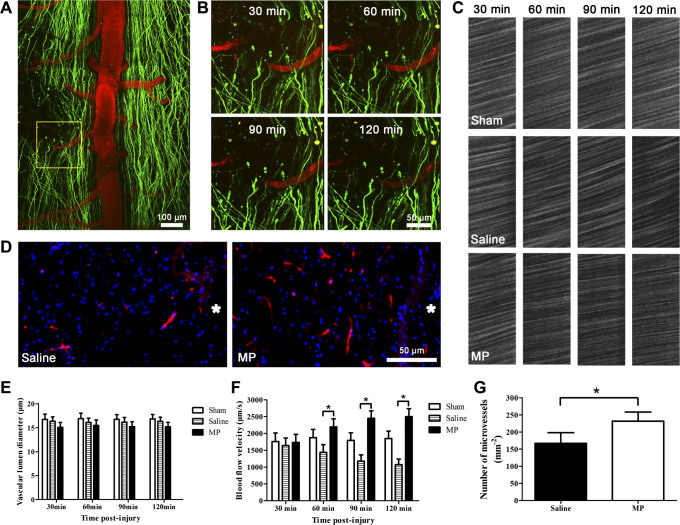
MP increased regional microvascular blood flow and reduced microvessel loss after SCI. (A) Representative image showing axons (green) and blood vessels (red) after hemisection SCI. The yellow square denotes: the monitored microvessel at the edge of lesion site. (B) In vivo two-photon imaging of a microvessel proximal to the lesion site at 30 min, 60 min, 90 min and 120 min post-injury. (C) In vivo two-photon imaging of regional microvascular blood flow from the sham group, saline-treated SCI group and the MP-treated SCI group at 30 min, 60 min, 90 min and 120 min post-injury. (D) Representative images depict blood vessels (red) at the edge of lesion sites in saline-treated group and MP-treated group. Nuclei were stained with DAPI (blue). Asterisk indicates lesion site. (E) Changes of vascular lumen diameter in the sham group (n = 6), saline-treated group (n = 6), and MP-treated group (n = 6) at 30 min, 60 min, 90 min and 120 min post-injury. P > 0.05. (F) Changes of microvascular blood flow velocity in the sham group (n = 6), saline-treated group (n = 6), and MP-treated group (n = 6) at 30 min, 60 min, 90 min and 120 min post-injury. Values presented are mean ± SEM. *P < 0.01. Repeated measure ANOVA followed by Fisher's LSD. (G) The number of microvessels at the edge of lesion site in saline-treated (n = 6) and MP-treated (n = 6) mice. Values presented are mean ± SEM. *P < 0.01. Student's *t* test.

We used *in vivo* two-photon imaging of microvessels proximal to the lesion site (Fig. 3A) to measure microvascular blood flow velocity and vascular lumen diameter at different time point (Fig. 3B) and investigate the effect of MP treatment after SCI^18^,^19^. Our results showed that the blood flow velocity in the sham group (n = 6) remained stable during all imaging sessions after surgery (Figs 3C and 3F). The regional spinal cord blood flow velocity decreased progressively after hemisection SCI in the saline-treated SCI group. The respective microvascular blood flow velocity at 30 min, 60 min, 90 min, and 120 min post-injury were 1635.01 ± 568.47 μm/s, 1435.77 ± 566.32 μm/s, 1175.82 ± 455.23 μm/s, and 1074.92 ± 399.64 μm/s in the saline-treated SCI group (n = 6). However, the regional spinal cord blood flow exhibited a sustained increase post-injury in the MP-treated SCI group. The respective microvascular blood flow velocity at 30 min, 60 min, 90 min, and 120 min post-injury were 1734.35 ± 583.99 μm/s, 2192.54 ± 593.66 μm/s, 2452.28 ± 535.59 μm/s, and 2499.34 ± 579.88 μm/s in the MP-treated SCI group (n = 6). The regional spinal cord blood flow velocity was significantly higher in the MP group than in the saline group (Fig. 3F, P < 0.05). However, the vascular lumen diameter in all groups exhibited no significant changes at 30 min, 60 min, 90 min, and 120 min post-injury (P > 0.05, Fig. 3E). We also examined the number of microvessels at the edge of lesion site at 3 days post-injury (Fig. 3D). The number of microvessels were 167.2 ± 12.65 vessels/mm^2^ in the saline-treated group (n = 6), and 231.8 ± 10.86 vessels/mm^2^ in the MP-treated group (n = 6). The saline group exhibited greater blood vessel loss than the MP group (Fig. 3G, P = 0.008). These results suggest that MP treatment can ameliorate microcirculation by increasing regional microvascular blood flow and reducing microvessels loss, which may contribute to the attenuation of progressive axonal damage and neuronal death.

### MP reduced calcium influx and the expression of active calpain-1 and cleaved caspase-3

**Figure 4:**
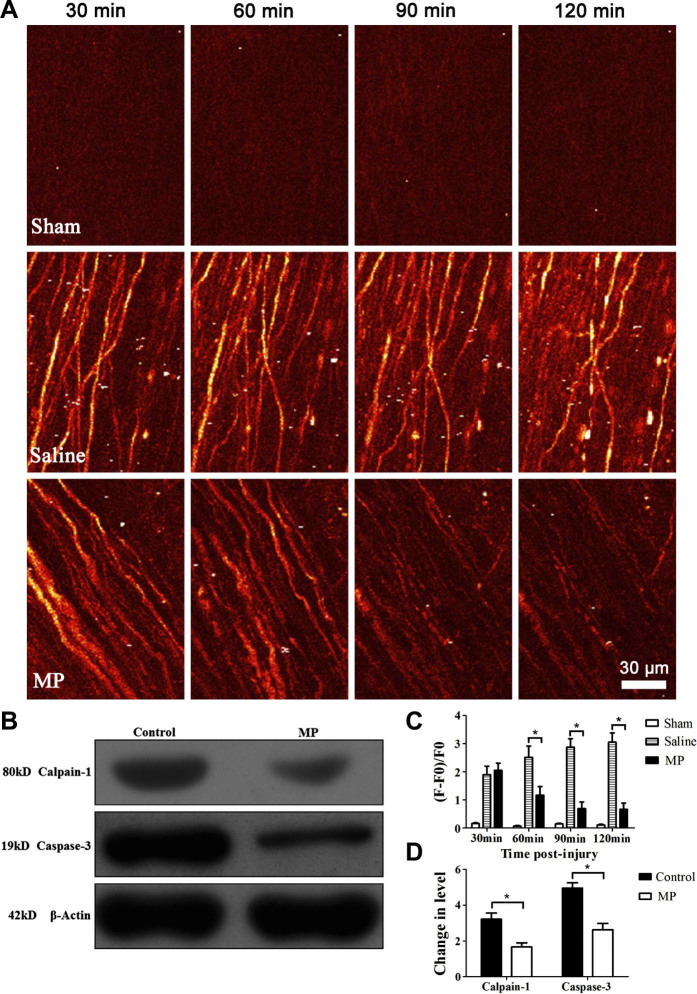
MP treatment reduced calcium influx and the expression of active calpain-1 and cleaved caspase-3 after SCI. (A) In vivo two-photon imaging of calcium influx in the sham group and injured axons in the saline-treated SCI group and the MP-treated SCI group at 30 min, 60 min, 90 min and 120 min post-injury. (B) Western blots of calpain-1 and cleaved caspase-3 expression in injured spinal cord segment of saline-treated group and MP-treated group. (C) Changes of calcium influx after hemisection SCI in sham group (n = 6), saline-treated (n = 6) and MP-treated (n = 6) mice at 30 min, 60 min, 90 min and 120 min post-injury. Values presented are mean ± SEM (P < 0.05). Statistical analysis: repeated measure ANOVA. Fifteen to twenty axons were measured individually per animal. (D) Quantification of protein band density to determine levels of active calpain-1 and cleaved caspase-3. *P < 0.01. Student's *t* test.

We used two-photon microscopy and Thy1-GCaMP transgenic mice to image the level of intracellular calcium [Ca^2+^]_i_ with a genetically-encoded calcium indicator GCaMP in injured axons in order to assess the effect of MP on calcium influx after SCI (Fig. 4A). Changes in [Ca^2+^]_i_ were expressed as changes in fluorescence intensity^20^. The level of [Ca^2+^]_i_ in the sham group (n = 6) remained low and had no significant change at 30 min, 60 min, 90 min, and 120 min post-surgery (P > 0.05). Changes in the level of [Ca^2+^]_i_ fluorescence in the saline group (n = 6) at 30 min, 60 min, 90 min, and 120 min were 1.89 ± 0.73, 2.51 ± 0.97, 2.87 ± 0.74, 3.05 ± 0.81 respectively. However, changes in the level of [Ca^2+^]_i_ fluorescence in the MP group (n = 6) at 30 min, 60 min, 90 min, and 120 min were 2.05 ± 0.62, 1.17 ± 0.74, 0.69 ± 0.58, 0.66 ± 0.54 respectively. The [Ca^2+^]_i_ in MP group were significantly lower than the saline group at 60 min, 90 min, and 120 min post-injury (Fig. 4C, P < 0.05). In order to assess the expression of calpain-1 gene and its apoptotic pathways that are downstream of increased [Ca^2+^]_i_, we measured changes in active calpain-1 and cleaved caspase-3 post-injury by Western blots analysis (Fig. 4B). Compared with saline treatment, MP treatment down-regulated the expression of calpain-1 and active caspase-3 in injured spinal cord segments (Fig. 4D, P < 0.05).

### MP inhibited the accumulation of microglia/macrophages, and down-regulated the expression of iNOS, MCP-1, and IL-1β

**Figure 5:**
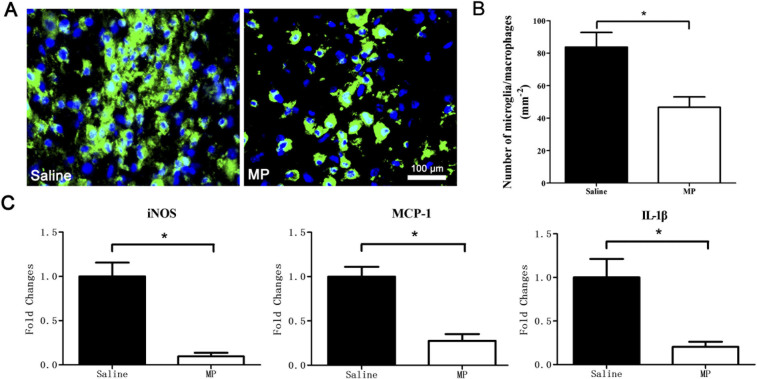
MP inhibited the accumulation of microglia/macrophages, and down-regulated the expression of iNOS, MCP-1, and IL-1β after SCI. (A) Representative F4/80 (green) and DAPI (blue) staining depicts the effects of MP on microglia/macrophages at the lesion site in saline-treated SCI group and MP-treated SCI group. (B) The number of microglia/macrophages at the lesion sites of saline-treated (n = 6) and MP-treated (n = 6) mice. Values presented are mean ± SEM. *P < 0.01. Student's *t* test. (C) Relative changes (quantitative real-time PCR) of selected proinflammatory markers including nitric oxide synthase (iNOS), monocyte chemoattractant protein 1 (MCP-1) and interleukin 1 beta (IL-1β) in the injured spinal cord segments from saline-treated (n = 5) and MP-treated (n = 5) mice. *P < 0.01. Student's *t* test.

We examined the number of microglia/macrophages, and MP's effect on their accumulation at the lesion sites 3 days post-injury (Fig. 5A). The number of microglia/macrophages at the lesion site was 83.69 ± 9.06 cells/mm^2^ in the saline-treated SCI group (n = 6) and 46.67 ± 6.41 cells/mm^2^ in the MP-treated SCI group (n = 6). The number of microglial/macrophages was greater in the saline group than in the MP group (Fig. 5B, P = 0.007). To evaluate the anti-inflammatory effect of MP in injured spinal cord, we performed a quantitative analysis of well-known proinflammatory markers iNOS, MCP-1, IL-1β in injured mouse spinal cord removed 72 h post-injury. Strong reductions of all tested markers were observed in the MP group (n = 5) compared with the saline group (n = 5). iNOS expression was reduced 10.3 fold, MCP-1 expression was reduced 3.6 fold, and IL-1 expression was reduced 4.9 fold. (Fig. 5C, P < 0.01 for all).

### MP improved the recovery of behavioral function

**Figure 6:**
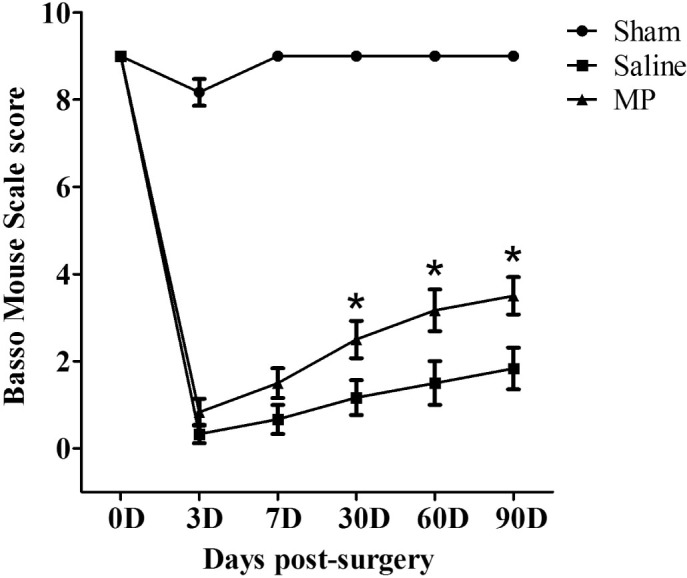
MP improved the recovery of behavioral function after SCI. Basso Mouse Scale (BMS) scores of mice in the sham group (n = 6), saline-treated group (n = 6), and MP-treated group (n = 6) at different time points (0 D, 3 D, 7 D, 30 D, 60 D, 90 D) post-surgery. The BMS scores in MP-treated group were higher than saline-treated group from 30 D to 90 D post-injury. Values presented are mean ± SEM. *P < 0.05. Repeated measure ANOVA followed by Fisher's LSD.

To evaluate the effects of MP in behavioral function after SCI, Basso Mouse Scale (BMS) was used to assess functional improvement of all groups at different time points (0 D, 3 D, 7 D, 30 D, 60 D, 90 D) after surgery (Fig. 6). The mice in the sham group (n = 6) exhibited mild trunk instability (BMS score 8) on day 3 post-surgery and recovered to normal trunk stability from day 7 onward (BMS score 9). The mice in the saline-treated group and MP-treated group exhibited no ankle movement and complete hind limb paralysis after hemisection SCI (BMS score 0). A few mice were capable of slight ankle movement during D7 post-injury in saline and MP groups, but there was no significant difference in BMS score (P > 0.05). The respective BMS scores at 30D, 60D, and 90D post-injury were 1.17 ± 0.98, 1.51 ± 1.22, 1.83 ± 1.16 in the saline-treated SCI group (n = 6), and 2.51 ± 1.05, 3.16 ± 1.16, 3.50 ± 1.04 in the MP-treated SCI group (n = 6). The BMS score was significantly higher in MP-treated group than in saline-treated group from 30 D, 60 D, and 90 D after SCI (P < 0.05).

## Discussion

Spinal cord injury includes primary and secondary injury phases. The primary injury phase comprises immediate cell death and vascular dysfunction, and is followed by a delayed secondary injury phase that can last from hours to weeks. Secondary injury triggers a wide range of down-stream pathological events that aggravate the primary injury, and causes progressive cell damage that is not involved in the primary injury^21^. However, the early pathological changes of axonal dieback, blood flow, and calcium influx into axons *in vivo* after SCI remain unclear. To explore the pathogenic mechanism of SCI, we conducted this study to investigate the early pathological changes of axonal dieback, blood flow and calcium influx into axons *in vivo* after SCI.

As the standard effective therapeutic agent now in use for the clinical treatment of acute SCI, the glucocorticoid drug MP has been shown to alleviate secondary injury by decreasing inflammation and ischemic reaction, as well as by inhibiting lipid peroxidation^22^. However, high-dose MP can cause many side effects, including infection, pneumonia, bleeding, and femoral head necrosis, and thus increase the risk of death^23^,^24^. In addition, some retrospective cohort studies have shown no differences in neurological outcome between SCI patients with or without MP therapy^14^. The use of high-dose MP in SCI patients is controversial on the basis of the risk of serious adverse effects and modest neurological benefit. In clinical treatment for SCI patients, MP is recommended to be administered within 8 h post-injury^25^. Previous study indicated that MP therapy on SCI model had a very short therapeutic window, the delayed treatment of MP showed no effect compared to the saline-treated group^26^. In the present study, MP was initially administered at 30 min post-injury and continuous administered at 6 h and 24 h to provide an effective concentration during the first day after SCI. Our study confirmed that the early application of MP was effective at reducing the post-SCI damage during the early stage and improved functional recovery at the later stage. These results consisted with previous study that MP treatment improved axonal survival and sprouting in complete transection SCI model^27^.

Previous laboratory studies of SCI were mostly confined to vitro experimental techniques, including tissue sectioning, immunohistochemistry, and BDA labeling^28^. These methods do not allow us to determine dynamic changes in the same animal over multiple days after SCI. The *in vivo* imaging techniques used in the past include MRI, micro-CT, diffusion tensor tractography^29^, any of which can be used to examine the same animal for a couple of days. However, these methods lack resolution at the micrometer level. Recent, two-photon microscopy has been used to examine pinprick-induced or laser-induced SCI models^30^,^31^,^32^,^33^,^34^. These models are able to control the damage in axons without damaging the neighboring neurons and vessels. In the present study, we used a hemisection SCI model, in which axons, neurons, and vessels can be damaged. We also modified a spinal stabilization device and implanted window that reduces the movement artifacts caused by heartbeat and breathing (Fig. 1), allowing us to examine axonal dieback, regional microvascular blood flow, and calcium influx into axons in the same animal for multiple hours. This *in vivo* imaging method allows us to evaluate the early dynamic changes and MP's effect after SCI in a less invasive manner.

Although previous study showed that MP therapy may reduce lesion volume after SCI^35^, the mechanisms underlying MP therapy remain unclear. In the present study, we conducted two-photon microscopy and employed YFP H-line transgenic mice to trace axonal dieback after hemisection SCI. Our results indicated that the axons in the sham group remained intact during all imaging sessions post-surgery. This finding indicated that the window implantation on the spinal cord did not cause significant damage to the axons. In the hemisection SCI groups, MP treatment reduced axonal dieback distance at 48 h post-injury when compared with the saline-treated mice. The histology revealed that the MP group also had a higher neuronal number than the saline group. In addition, MP improved the functional recovery at the later stage of SCI. These findings suggest that MP therapy may help attenuate progressive axonal damage and neuronal death, improve neurological recovery after SCI. These findings supported the idea that the early application of MP improved the neuronal viability and promoted neurite outgrowth after SCI^36^,^37^.

Previous studies often used Doppler ultrasound to evaluate the blood flow after SCI^4^. It is difficult to detect the microvascular blood flow at the edge of injured epicenter using this method. In this study, we conducted *in vivo* two-photon imaging of microvessels of 10–20 μm diameter labeled with Texas Red dextran and measured the blood flow velocity for several hours post-injury. Our results revealed that the microvascular blood flow velocity and vascular lumen diameter in the uninjured sham group remained stable during all imaging sessions after surgery, this finding suggested that the implanted window on the spinal cord did not cause significant damage to the microvessels and the blood flow. In addition, the microvascular blood flow velocity in saline-treated group decreased progressively post-injury. Thrombus, and dysfunction of vascular homeostasis might be important contributors to this event^38^. However, microvascular blood flow velocity was significantly increased in the MP-treated group compared with the saline-treated group. These results consisted with the previous findings that MP treatment after SCI improved microvascular perfusion^39^. However, the vascular lumen diameter in all groups exhibited no significant changes at all imaging sessions post-injury. Thus, the increase blood flow is not due to vasospasm and vasodilatation-induced hyperemia in the monitored venules. Histology also showed that MP-treated mice had a higher microvessels number at the edge of lesion site than saline-treated mice, which suggests that high-dose MP treatment reduces microvessels loss after SCI.

The initial trauma in the spinal cord disrupts the cell membrane and axolemma, leading to a sudden influx of extracellular calcium. It also causes mitochondrial damage that can affect Na-K-ATPase activity, as well as an increase of intracellular calcium via dysfunction of Na-Ca-exchanger^40^. The intracellular calcium concentration activates the calcium-activated neutral proteinase calpain-1, which results in neuronal disintegration and apoptosis^41^. These are essential pathogenic factors in the secondary phase of SCI. To understand how MP affects calcium influx in injured axons, we used Thy1-GCaMP transgenic mice, which express genetically encoded calcium indicators in neurons and axons. Two-photon microscopy was used to image the calcium influx in injured axons post-injury. MP treatment produced a significant reduction of calcium influx compared with the saline-treated group post-injury. The expression of active calpain-1 and cleaved caspase-3 were down-regulated in MP-treated mice compared with saline-treated mice. These findings may suggest that the membrane-stabilization effects of MP prevent excessive calcium influx into cells^42^. MP also reduced the expression of active calpain-1 and cleaved caspase-3 post-injury^43^. These changes of expression might be the important factors of how MP reduces secondary injury after SCI.

Microglia are the resident immune cells in the spinal cord. When traumatic damage is inflicted on the spinal cord, the blood-spinal cord barrier is damaged. Microglia/macrophages were recruited and accumulated at the lesion site after SCI and secreted proinflammatory cytokines that cause neuronal toxicity^44^,^45^,^46^. The proinflammatory mediators iNOS, MCP-1, and IL-1β are strongly associated with neurologic impairment. NO and ATP mediated the conversion of microglial shape from ramified to ameboid indicating cellular activation^33^. Activated microglia/macrophages induced axonal dieback through direct physical interactions^47^. In this study, we found that microglia/macrophages accumulated at the site of injury after SCI, and MP treatment inhibited the accumulation of microglia/macrophages, down-regulated the expression of iNOS, MCP-1 and IL-1β. This also might be a major point of the mechanism underlying the beneficial neuroprotective effect of MP in this model of acute SCI.

In conclusion, our data demonstrate that MP exerts a protective effect during the early stages of hemisection SCI in this mouse model. Our findings are consistent with previous studies that MP therapy may alleviate the progressive damage of axons and reduce accumulation of microglia/macrophages^48^. However, we used hemisection injury model rather than compression injury model. The difference of the injury model might be a major reason which caused different results. In addition, we observed a longer period to assess functional improvement of the animals and found that MP treatment improved the recovery of behavioural function after SCI. Our results further suggest that MP increase microvascular blood flow and reduce microvessel loss, reduce calcium influx and down-regulate the expression of active calpain-1 and cleaved caspase-3, and down-regulate the expression of iNOS, MCP-1 and IL-1β. These findings suggest that early application of MP may be an effective treatment for acute SCI.

Lastly, it is important to point out some limitations of our studies related to repeated imaging with *in vivo* two-photon microscopy. There was mild inflammatory responses caused by the implanted window as previous described by Farrar and our preliminary experiment^49^. Previous studies showed that even a minimal injury to the spinal cord caused enormous increase in microglia number and density around the lesion site. However, this increase far exceeded the microglia response caused by implanted window. This moderate inflammatory reaction does not seem to significantly affect the results caused by MP treatment after SCI. In present study, all animals were treated in the same condition and experienced the same model, this could help to minimize the variance between groups. In addition, the two-photon microscopy can only image axons less than 200 μm deep in the dorsal columns, it is difficult to image the deeper tissue in live mouse spinal cord. The growth of granulation tissue also affect the quality of image^34^. Furthermore, there are a number of effects of MP on spinal cord injury treatment. It is not clear which of these is responsible for the therapeutic effect. Further research needs to address these issues.

## Methods

### Animals

Animal surgical procedures were conducted with the approval of the Animal Experimentation Ethics of the Chinese PLA General Hospital. All experiments were carried out in accordance with Animal Experimentation Ethics Guidelines of the Chinese PLA General Hospital. Animals had free access to food and water. Two lines of transgenic mice, the YFP H-line and the Thy1-GCaMP line (male, 8–10 weeks of age, 20–25 g) were used in this study. YFP-H line mice specifically expressed yellow fluorescent protein (YFP) in motor and sensory neurons and axons^50^. Thy1-GCaMP transgenic mice expressed a genetically encoded GFP-based calcium indicator protein in motor and sensory neurons and axons^17^,^51^. We implanted the glass window after laminectomy or hemisection injury to the spinal cord. Then we randomly divided each mouse line into three groups (n = 6 mice per subgroup). The sham group, the saline-treated SCI group and the MP-treated SCI group each included YFP-H line mice (n = 6 per group) and Thy1-GCaMP mice (n = 6 per group). The sham group received laminectomy only. The saline-treated SCI group received saline intraperitoneally at 30 min, 6 h, 24 h after SCI. The MP-treated SCI group received MP intraperitoneally at 30 min, 6 h, 24 h after SCI. MP was administered at doses of 30 mg/kg, as recommended by the National Acute Spinal Cord Injury Study (NASCIS) 2, 3 trials and as reported previously^13^,^25^,^52^. The criteria for animal exclusion. During the surgery process, two YFP-H line mice died due to inappropriate anesthesia, we added other two YFP-H line mice (male, 8–10 weeks of age, 20–25 g) and randomly divided into the groups.

### SCI model and implantation of the imaging window

We performed all surgical procedures with special attention to sterile conditions. 20 mg/ml ketamine and 2 mg/ml xylazine were administered intraperitoneally to anesthetize the mice. For each moue, the dorsal surface above the thoracic spinal region was shaved with an electric razor and washed with 70% (v/v) ethanol and iodine to reduce the risk of infection. We made a longitudinal incision in the skin at the T11-T13 level of the spine and removed the muscle and tendon tissue from the spinal arcs. After the laminectomy at the level of the T12 segment, we used a sharp scalpel to make a hemisection injury in the spinal cord as previous reported^53^,^54^. Briefly, we used stainless clamps of stereotaxic apparatus to immobilize the spinal column, then we used microsurgical forceps and microscissor to tear the dura of the spinal cord segment. A sharp scalpel of 150 μm width was used to cut to the ventral cord on the middle of the spinal cord, and then transected the whole left spinal cord to the lateral side^55^. The average width of the induced injury was 160.8 ± 7.3 μm. All the surgery procedure were performed under the stereomicroscope. The surgical manipulation is very reproducible and all the SCI surgeries in present study were performed by the same experienced operator, this could also help to minimize the variation of the lesion size. Because the bleeding was a serious concern for two-photon imaging process, we avoided to damage the dorsal central vein in this model. However, there was bleeding from the injured microvessels after hemisection spinal cord injury. In order to avoid the influence of blood in two-photon imaging process, we cleared the blood from the injured spinal cord by flushing the exposed cord with sterile PBS. After clearing the blood from the lesion site, we implanted a glass window on the mouse spinal cord according to previously described methods^49^. Briefly, we used two metal bars to clamp the three vertebrae on either side of the laminectomy, put the top plate onto the metal bars, and sealed the bone and bars with cyanoacrylate and dental acrylic. Then we applied a layer of silicone elastomer over the spinal cord and placed a glass coverslip over the spinal cord. Finally, we glued the window with dental cement and sutured the skin to the top plate (Fig. 1). The process of window implantation took 23.4 ± 3.5 min after the hemisection injury by an experience operator. After the operation of injury model and window implantation, we randomly divided the animals into different groups without knowing the exact size of the injury and then took the animals to the two-photon microscopy for the first imaging session, we set the 30 min post-injury as the first imaging time in all groups. Postoperatively, mice were kept in a warning pad for several hours until they regained consciousness. We manually voided the bladders of the mice twice daily until voluntary control returned^56^. An antibiotic (enrofloxacin, 2.27 mg/kg, Baytril, Bayer, KS, USA) was used once daily for 3 days. The mice had free access to food and tap water and were maintained on a 12 h light/dark cycle at 22°C ± 1°C.

### In vivo imaging of axonal dieback, regional microvascular blood flow, and calcium influx into axons after SCI

To reduce motion artifacts, we positioned each mouse in a customized spinal stabilization device and slightly elevated it to allow room for breathing and chest expansion, as previously described^57^. We used an Olympus FluoView FV1000 two-photon microscope with an Olympus 25 × 1.0 NA water-immersion objective lens. A Spectra Physics Mai-Tai IR laser was tuned to 920 nm for two-photon excitation of YFP and to 890 nm for calcium imaging. Each mouse was kept warm at 37°C during the imaging period. The axonal dieback was a relatively slower event, so we selected the time point at 30 min, 8 h, 24 h, and 48 h post-injury to perform two-photon imaging studies, with the blood vessels labeled with Texas Red dextran (70 kDa) as previous described^58^. Fifteen to twenty axons were measured per animal. We imaged the regional microvascular blood flow as previous reported^59^. After injection of Texas Red dextran into the tail vein, we first mapped the vasculature at the edge of lesion site with a 25 × 1.0 NA water-immersion objective lens. Changes in blood flow and calcium influx was most drastic in the first 2 h post-injury, so we selected the time point at 30, 60, 90, and 120 min post-injury to detect these events with the hope to detect the effect of our pharmacological manipulation. We monitored microvessels of 10–20 μm diameter within 200 μm of the lesion site. Linear scanning along the length of the center of each microvessel was used to measure the velocity of Red Blood Cells (RBCs) at 30 min, 60 min, 90 min, and 120 min post-injury. The RBCs velocity was calculated from the angle of the RBCs streaks^60^. The vascular lumen diameter was measured by the width of the vessels at 30 min, 60 min, 90 min, and 120 min post-injury^18^. For calcium imaging, we used Thy1-GCaMP transgenic mice to investigate intracellular calcium levels in injured axons at 30 min, 60 min, 90 min, and 120 min after SCI. The laser power at the back aperture of the objective was 30 mW at 900 nm at specimen, and the power was constant during all imaging sessions. We measured the fluorescence intensity changes in intracellular calcium levels to evaluate the calcium influx post-injury. Values presented are mean ± SEM. Repeated measure ANOVA followed by Fisher's LSD.

### Image processing and quantification

Image analysis was performed using NIH Image J software. We pseudo-colored and enhanced the contrast of images to increase clarity. We traced the dieback of individual axons in the caudal area. We tracked axons from both three-dimensional stacks to determine the distance between individual axon tips from the initial lesion site. Fifteen to twenty axons were measured per animal to determine the average axonal dieback distance from the edge of the observed injury^49^. The measurements from all animals in each group were averaged to yield the average dieback distance per time point. To evaluate changes of the regional microvascular blood flow velocity, we measured the velocity of RBCs with linear scanning along the length of center of each microvessel, and then calculated the angle of the RBCs streaks. To evaluate calcium influx in the injured axons, we imaged the calcium fluorescence intensity at injured axons in Thy1-GCaMP mice. Changes in [Ca^2+^]_i_ were expressed as changes in fluorescence intensity. (F―F0)/ F0 was used where we defined F as the fluorescence in single axon and F0 as the resting fluorescence signal^20^. Fifteen to twenty axons 200 μm away from the lesion edge were measured individually per animal.

### Histology

We performed a histological analysis at the lesion site 3 days post-injury. Animals were deeply anesthetized and perfused transcardially with 20 ml PBS solution, followed by fixation with 20 ml 4% paraformaldehyde (PFA). We immersed the entire spine in 4% PFA for 1 day and then removed the spinal cord from the vertebral canal with microsurgical scissors. The spinal cord was immersed in 30% sucrose until saturated and embedded into optimal cutting temperature (OCT) compound. We froze the spinal cord at g−80°C overnight and cut 10 μm sections on a Microm HM 525 cryotome (ThermoFisher Scientific). We blocked with a mixture of 2% goat serum in PBS for 1 h. Next, sections were incubated overnight at 4°C with a primary anti-MAP2 IgG antibody (1:100 dilution; Millipore, USA), anti-F4/80 IgG antibody (1:100 dilution; Biolengend, USA). After incubation with the primary antibody, we rinsed tissue sections in PBS and incubated them with FITC-conjugated anti-mouse fluorescent secondary antibodies (1:100 dilution) for 2 h, and then incubated with DAPI at room temperature for 20 min. Blood vessels were directly labeled by dye DiI (Sigma, USA) as previous reported^61^. Briefly, 100 mg of DiI crystal was dissolved in 16.7 ml of 100% ethanol. After deeply anesthetized the mice, we perfused the mice transcardially with 5 ml DiI solution at a rate of 1–2 ml/min. In this method, DiI molecules were directly incorporated into the membrane of endothelial cells. After DiI staining the blood vessels, the spinal cord was fixed and cut sections as above described. We examined the sections with a fluorescence microscope. Neuronal somata were manually counted based on the morphology of neuron and counter-staining of MAP-2 antibody (red) as well as DAPI (blue). Microvessels were manually counted based on the counter-staining of DiI (red) as well as DAPI (blue). We counted the neurons and microvessels in two rectangular area (0.39 mm^2^) at the edge of lesion site per section. Three sections per mouse were quantified. Microglia/macrophages were manually counted based on the morphopogy of microglia/macrophages and counter-staining of F4/80 antibody (green) and DAPI (blue). Microglia/macrophages were counted in a rectangular area (5.07 mm^2^) in the middle of lesion site per section. Three sections per mouse were quantified. Six mice in each group were used.

### Protein extraction and Western bolt analysis

Western blots was performed as reported previously^62^. We harvested and froze the injured spinal cord tissue at −70°C and then homogenized the tissue in buffer containing 50 μM Tris-HCl (pH = 7.4), 1 mM phenylmethysulfonyl (PMSF; Bethesda Research Laboratories, Gaithersburg, MD, USA) and 5 mM EGTA (Sigma) and homogenized with a Polytron batch homogenizer. We centrifuged the homogenized samples in an Optima LE-80K Ultracentrifuge (Beckman Coulter, Fullerton, CA, USA) for 1 h at 100,000 g. After centrifugation, we mixed protein samples with sample buffer and then boiled for 5 min and stored at −20°C. We loaded the samples onto 20% gels and electrophoresed at 200 V for 30 min. We then resolved the proteins and used a Genie transfer apparatus to transfer the samples to nylon membrane. We blocked the nylon membrane for 1 h in 5% nonfat milk in Tris/Tween buffer. We incubated the membranes overnight with primary IgG antibody (1:5000 anti-β-actin (clone AC-15; Sigma), 1:500 anti-active calpain-1 (Abcam, USA), and 1:500 anti-cleaved caspase-3 (Cell Signaling, USA)). We incubated the membranes with donkey anti-rabbit secondary antibody (diluted 1:2000; Biolegend, USA) for 1 h after washing three times with Tris/Tween buffer. We then incubated the membranes with chemiluminescent (ECL) reagent (Amersham, Piscataway, NJ, USA) and exposed them to X-Omat AR films (Kodak, Rochester, NY, USA). We scanned the films on a Umax PowerLook Scanner and used Photoshop software (Adobe Systems, Seattle, WA, USA) for image processing. We used Quantity One software (Bio-Rad) to determine the optical density (OD) of each band^40^,^62^.

### RNA extraction and real-time PCR analysis

We used the RNeasy Mini Kit (Qiagen, Germantown, MD, USA) to isolate the total mRNA from injured spinal cord segments (1 cm containing and surrounding the lesioned area) 3 days after SCI. One milliliter Trizol (Life Technologies) was used to homogenize the tissues, and RNA was extracted according to the manufacturer's protocol. We synthesized cDNA from 1 μg total RNA using iScript cDNA synthesis Kit (Bio-Rad, Hercules, CA, USA) after treatment with DNase (Promega, Madison, WI, USA). We used SYBR-Green based technology to perform real-time PCR in the CFX Connect Real-Time PCR Detection System (Bio-Rad); the following primers were used: nitric oxide synthase 2 (iNOS2) (Fw: AAACCCCAGGTGCTATTCCC; Rv: GAACATTCTGTGCAGTCCCA); monocyte chemoattractant protein 1 (MCP-1):( Fw: ACGCTTCTGGGCCTGTTGTT; Rv: CCTGCTGCTGGTGATTCTCT); Interleukin-1 beta (IL-1β): (Fw: TGGCAACT GTCCCTGAACTC; Rv: GTCGAGATGCTGCTGTGAGA). We analyzed the data using Bio-Rad CFX Manager 3.0 (Bio-Rad). The gene glyceraldehyde-3-phosphate dehydrogenase (GADPH) was chosen as reference. The mRNA level of each target gene was normalized by GADPH and expressed as 2^ΔCt^ (ΔCt = Ct target -Ct GADPH). The relative quantity in mRNA levels of tested genes was determined by the equation: relative quantity = 1000/2^ΔCt^.

### Behavioral testing

We used the Basso Mouse Scale (BMS) score to assess functional recovery after SCI as previously described^63^. Hind limb motor function was assessed with the 10-point scale in an open field. No ankle movement and complete hind limb paralysis scored 0; Slight ankle movement scored 1; Mild trunk instability scored 8 and no locomotor deficits scored 9. We assessed and scored the functional improvement of all groups on 0 D, 3 D, 7 D, 30 D, 60 D, 90 D after surgery. All experiments were performed in a double-blind manner. Values presented are mean ± SEM. Repeated measure ANOVA followed by Fisher's LSD.

### Statistical analysis

The statistical analysis was performed using SPSS (version17, SPSS IL, Chicago). Data are presented as mean ± SEM. We compared axonal dieback distance, microvascular blood flow, vascular lumen diameter, and calcium influx into axons using repeated measure ANOVA followed by Fisher's LSD. We compared number of neurons, microvessels, protein expression, microglia/macrophages, and inflammatory factors using Student's *t* test. Significant differences were defined at P < 0.05. Highly significant differences were defined at P < 0.01.

### Ethical statement

Animal surgical procedures were conducted with the approval of the Animal Experimentation Ethics of the Chinese PLA General Hospital. Care was taken to minimize the number of animals used and their suffering.

## Author Contributions

L.Z, W-B.G, S.Z, and Z.H. conceived the experiments. P.T, Y.Z, C.C, and X.J performed the experiments. F.J prepared the figures. X.L and W.L analyzed the data. Y.Z wrote the manuscript. All authors discussed the results and commented on the manuscript.

## Additional information

**How to cite this article**: Tang, P. *et al.* In Vivo Two-Photon Imaging of Axonal Dieback, Blood Flow, and Calcium Influx with Methylprednisolone Therapy after Spinal Cord Injury. *Sci. Rep.* **5**, 9691; DOI:10.1038/srep09691 (2015).

## References

[b1] Bracken M. B. Steroids for acute spinal cord injury. Cochrane Db Syst Rev 1, CD001046 (2012). 10.1002/14651858.CD001046.pub2 PMC6513405 22258943

[b2] Sekhon L. H. & Fehlings M. G. Epidemiology, demographics, and pathophysiology of acute spinal cord injury. Spine 26, S2–12 (2001). 11805601 10.1097/00007632-200112151-00002

[b3] Cerqueira S. R. *et al.* Microglia Response and In Vivo Therapeutic Potential of Methylprednisolone-Loaded Dendrimer Nanoparticles in Spinal Cord Injury. Small 9, 738–49 (2012). 23161735 10.1002/smll.201201888

[b4] Guizar-Sahagun G. *et al.* Glutathione monoethyl ester improves functional recovery, enhances neuron survival, and stabilizes spinal cord blood flow after spinal cord injury in rats. Neuroscience 130, 639–49 (2005). 15590148 10.1016/j.neuroscience.2004.09.056

[b5] Soubeyrand M. *et al.* Rat model of spinal cord injury preserving dura mater integrity and allowing measurements of cerebrospinal fluid pressure and spinal cord blood flow. Eur Spine J 22, 1810–19 (2013). 23508337 10.1007/s00586-013-2744-2 PMC3731496

[b6] Figley S. A. *et al.* A spinal cord window chamber model for in vivo longitudinal multimodal optical and acoustic imaging in a murine model. PloS One 8, e58081 (2013). 23516432 10.1371/journal.pone.0058081 PMC3597636

[b7] Momeni H. R. Role of calpain in apoptosis. Cell J 13, 65–72 (2011). 23507938 PMC3584455

[b8] Hohlfeld R., Kerschensteiner M. & Meinl E. Dual role of inflammation in CNS disease. Neurology 68, S58–63 (2007). 17548571 10.1212/01.wnl.0000275234.43506.9b

[b9] Gelosa P. *et al.* Microglia is a key player in the reduction of stroke damage promoted by the new antithrombotic agent ticagrelor. J Cerebr Blood F Met 34, 979–88 (2014). 10.1038/jcbfm.2014.45 PMC4050242 24643079

[b10] Suehiro K. *et al.* Ecto-domain phosphorylation promotes functional recovery from spinal cord injury. Sci Rep 4, 4972 (2014). 24826969 10.1038/srep04972 PMC4021324

[b11] Scali M. *et al.* Fluoxetine treatment promotes functional recovery in a rat model of cervical spinal cord injury. Sci Rep 3, 2217 (2013). 23860568 10.1038/srep02217 PMC3713566

[b12] Hall E. D. & Braughler J. M. Acute effects of intravenous glucocorticoid pretreatment on the in vitro peroxidation of cat spinal cord tissue. Exp Neurol 73, 321–324 (1981). 7250287 10.1016/0014-4886(81)90067-4

[b13] Bracken M. B. *et al.* A randomized, controlled trial of methylprednisolone or naloxone in the treatment of acute spinal-cord injury. Results of the Second National Acute Spinal Cord Injury Study. New Engl J Med 322, 1405–1411 (1990). 2278545 10.1056/NEJM199005173222001

[b14] Felleiter P., Muller N., Schumann F., Felix O. & Lierz P. Changes in the use of the methylprednisolone protocol for traumatic spinal cord injury in Switzerland. Spine 37, 953–6 (2012). 22020592 10.1097/BRS.0b013e31823a07a2

[b15] Anderson K. D., Sharp K. G. & Steward O. Bilateral cervical contusion spinal cord injury in rats. Exp Neurol 220, 9–22 (2009). 19559699 10.1016/j.expneurol.2009.06.012 PMC2761499

[b16] Zhang Y., Hou S. & Wu Y. Changes of intracellular calcium and the correlation with functional damage of the spinal cord after spinal cord injury. Chinese J Traumatol 5, 40–42 (2002). 11835756

[b17] Chen Q. *et al.* Imaging neural activity using Thy1-GCaMP transgenic mice. Neuron 76, 297–308 (2012). 23083733 10.1016/j.neuron.2012.07.011 PMC4059513

[b18] Shih A. Y. *et al.* Two-photon microscopy as a tool to study blood flow and neurovascular coupling in the rodent brain. J Cerebr Blood F Met 32, 1277–1309 (2012). 10.1038/jcbfm.2011.196 PMC3390800 22293983

[b19] Kim T. N. *et al.* Line-scanning particle image velocimetry: an optical approach for quantifying a wide range of blood flow speeds in live animals. PloS One 7, e38590 (2012). 22761686 10.1371/journal.pone.0038590 PMC3383695

[b20] Mills L. R., Velumian A. A., Agrawal S. K., Theriault E. & Fehlings M. G. Confocal imaging of changes in glial calcium dynamics and homeostasis after mechanical injury in rat spinal cord white matter. NeuroImage 21, 1069–1082 (2004). 15006675 10.1016/j.neuroimage.2003.10.041

[b21] Hall E. D. & Springer J. E. Neuroprotection and acute spinal cord injury: a reappraisal. NeuroRx 1, 80–100 (2004). 15717009 10.1602/neurorx.1.1.80 PMC534914

[b22] Hall E. D. Antioxidant therapies for acute spinal cord injury. Neurotherapeutics 8, 152–167 (2011). 21424941 10.1007/s13311-011-0026-4 PMC3101837

[b23] Baptiste D. C. & Fehlings M. G. Update on the treatment of spinal cord injury. Prog Brain Res 161, 217–233 (2007). 17618980 10.1016/S0079-6123(06)61015-7

[b24] Failli V. *et al.* Functional neurological recovery after spinal cord injury is impaired in patients with infections. Brain 135, 3238–3250 (2012). 23100450 10.1093/brain/aws267

[b25] Bracken M. B. Steroids for acute spinal cord injury. Cochrane Db Syst Rev 3**,** CD001046 (2002). 10.1002/14651858.CD001046 12137616

[b26] Yoon D. H., Kim Y. S. & Young W. Therapeutic time window for methylprednisolone in spinal cord injured rat. Yonsei Med J 40, 313–320 (1999). 10487132 10.3349/ymj.1999.40.4.313

[b27] Oudega M., Vargas C. G., Weber A. B., Kleitman N. & Bunge M. B. Long-term effects of methylprednisolone following transection of adult rat spinal cord. Eur J Neurosci 11, 2453–2464 (1999). 10383635 10.1046/j.1460-9568.1999.00666.x

[b28] Liu K. *et al.* PTEN deletion enhances the regenerative ability of adult corticospinal neurons. Nat Neurosci 13, 1075–1081 (2010). 20694004 10.1038/nn.2603 PMC2928871

[b29] Takano M. *et al.* In vivo tracing of neural tracts in tiptoe walking yoshimura mice by diffusion tensor tractography. Spine 38, E66–72 (2013). 23124261 10.1097/BRS.0b013e31827aacc2

[b30] Dibaj P. *et al.* In Vivo imaging reveals distinct inflammatory activity of CNS microglia versus PNS macrophages in a mouse model for ALS. PloS One 6, e17910 (2011). 21437247 10.1371/journal.pone.0017910 PMC3060882

[b31] Fenrich K. K. *et al.* Long-term in vivo imaging of normal and pathological mouse spinal cord with subcellular resolution using implanted glass windows. J Physiol 590, 3665–75 (2012). 22641787 10.1113/jphysiol.2012.230532 PMC3476626

[b32] Kerschensteiner M., Schwab M. E., Lichtman J. W. & Misgeld T. In vivo imaging of axonal degeneration and regeneration in the injured spinal cord. Nat Med 11, 572–7 (2005). 15821747 10.1038/nm1229

[b33] Dibaj P. *et al.* NO mediates microglial response to acute spinal cord injury under ATP control in vivo. Glia 58, 1133–1144 (2010). 20468054 10.1002/glia.20993

[b34] Laskowski C. J. & Bradke F. In vivo imaging - A dynamic imaging approach to study spinal cord regeneration. Exp Neurol 242, 11–7 (2012). 22836145 10.1016/j.expneurol.2012.07.007

[b35] Kim Y. T., Caldwell J. M. & Bellamkonda R. V. Nanoparticle-mediated local delivery of Methylprednisolone after spinal cord injury. Biomaterials 30, 2582–2590 (2009). 19185913 10.1016/j.biomaterials.2008.12.077 PMC2678685

[b36] Okonkwo D. O. *et al.* A comparison of adenosine A2A agonism and methylprednisolone in attenuating neuronal damage and improving functional outcome after experimental traumatic spinal cord injury in rabbits. J Neurosurg-Spine 4, 64–70 (2006). 16506468 10.3171/spi.2006.4.1.64

[b37] Liu W. L. *et al.* Methylprednisolone inhibits the expression of glial fibrillary acidic protein and chondroitin sulfate proteoglycans in reactivated astrocytes. Glia 56, 1390–1400 (2008). 18618653 10.1002/glia.20706

[b38] Carlson G. D. *et al.* Sustained spinal cord compression: part II: effect of methylprednisolone on regional blood flow and recovery of somatosensory evoked potentials. J Bone Joint Surg Am 85-A, 95–101 (2003). 12533578

[b39] Anderson D. K., Means E. D., Waters T. R. & Green E. S. Microvascular perfusion and metabolism in injured spinal cord after methylprednisolone treatment. J Neurosurg 56, 106–113 (1982). 7054403 10.3171/jns.1982.56.1.0106

[b40] Samantaray S. *et al.* Low dose estrogen prevents neuronal degeneration and microglial reactivity in an acute model of spinal cord injury: effect of dosing, route of administration, and therapy delay. Neurochem Res 36, 1809–1816 (2011). 21611834 10.1007/s11064-011-0498-y PMC3162090

[b41] Hogan E. L., Hsu C. Y. & Banik N. L. Calcium-activated mediators of secondary injury in the spinal cord. Cent Nerv Syst Trauma 3, 175–179 (1986). 3533279 10.1089/cns.1986.3.175

[b42] Young W. & Flamm E. S. Effect of high-dose corticosteroid therapy on blood flow, evoked potentials, and extracellular calcium in experimental spinal injury. J Neurosurg 57, 667–673 (1982). 7131067 10.3171/jns.1982.57.5.0667

[b43] Buttgereit F., Krauss S. & Brand M. D. Methylprednisolone inhibits uptake of Ca2+ and Na+ ions into concanavalin A-stimulated thymocytes. Biochem J 326, 329–332 (1997). 9291100 10.1042/bj3260329 PMC1218673

[b44] Donnelly D. J. & Popovich P. G. Inflammation and its role in neuroprotection, axonal regeneration and functional recovery after spinal cord injury. Exp Neurol 209, 378–388 (2008). 17662717 10.1016/j.expneurol.2007.06.009 PMC2692462

[b45] Li T. *et al.* Proliferation of parenchymal microglia is the main source of microgliosis after ischaemic stroke. Brain 136, 3578–3588 (2013). 24154617 10.1093/brain/awt287

[b46] Greenhalgh A. D. & David S. Differences in the phagocytic response of microglia and peripheral macrophages after spinal cord injury and its effects on cell death. J Neurosci 34, 6316–22 (2014). 24790202 10.1523/JNEUROSCI.4912-13.2014 PMC6608120

[b47] Horn K. P., Busch S. A., Hawthorne A. L., van Rooijen N. & Silver J. Another barrier to regeneration in the CNS: activated macrophages induce extensive retraction of dystrophic axons through direct physical interactions. J Neurosci 28, 9330–9341 (2008). 18799667 10.1523/JNEUROSCI.2488-08.2008 PMC2567141

[b48] Yiling Z. *et al.* Two-Photon Excited Fluorescence Microscopy as a Tool to Investigate the Efficacy of Methylprednisolone in a Mouse Spinal Cord Injury Model. Spine 39, E493–9 (2014). 24480947 10.1097/BRS.0000000000000218

[b49] Farrar M. J. *et al.* Chronic in vivo imaging in the mouse spinal cord using an implanted chamber. Nat Methods 9, 297–302 (2012). 22266542 10.1038/nmeth.1856 PMC3429123

[b50] Feng G. *et al.* Imaging neuronal subsets in transgenic mice expressing multiple spectral variants of GFP. Neuron 28, 41–51 (2000). 11086982 10.1016/s0896-6273(00)00084-2

[b51] Zariwala H. A. *et al.* A Cre-dependent GCaMP3 reporter mouse for neuronal imaging in vivo. J Neurosci 32, 3131–3141 (2012). 22378886 10.1523/JNEUROSCI.4469-11.2012 PMC3315707

[b52] Nash H. H., Borke R. C. & Anders J. J. Ensheathing cells and methylprednisolone promote axonal regeneration and functional recovery in the lesioned adult rat spinal cord. J Neurosci 22, 7111–7120 (2002). 12177207 10.1523/JNEUROSCI.22-16-07111.2002 PMC6757894

[b53] Kanno H., Ozawa H., Sekiguchi A., Yamaya S. & Itoi E. Induction of autophagy and autophagic cell death in damaged neural tissue after acute spinal cord injury in mice. Spine 36, E1427–34 (2011). 21304420 10.1097/BRS.0b013e3182028c3a

[b54] Dong H. *et al.* Enhanced oligodendrocyte survival after spinal cord injury in Bax-deficient mice and mice with delayed Wallerian degeneration. J Neurosci 23, 8682–91 (2003). 14507967 10.1523/JNEUROSCI.23-25-08682.2003 PMC6740425

[b55] Kalderon N. & Fuks Z. Structural recovery in lesioned adult mammalian spinal cord by x-irradiation of the lesion site. Proc Natl Acad Sci U S A 93, 11179–11184 (1996). 8855329 10.1073/pnas.93.20.11179 PMC38304

[b56] Wu B. *et al.* Improved regeneration after spinal cord injury in mice lacking functional T- and B-lymphocytes. Exp Neurol 237, 274–285 (2012). 22868200 10.1016/j.expneurol.2012.07.016

[b57] Davalos D. *et al.* Stable in vivo imaging of densely populated glia, axons and blood vessels in the mouse spinal cord using two-photon microscopy. J Neurosci Meth 169, 1–7 (2008). 10.1016/j.jneumeth.2007.11.011 PMC2647134 18192022

[b58] Dray C., Rougon G. & Debarbieux F. Quantitative analysis by in vivo imaging of the dynamics of vascular and axonal networks in injured mouse spinal cord. Proc Natl Acad Sci U S A 106, 9459–9464 (2009). 19470644 10.1073/pnas.0900222106 PMC2685250

[b59] Zhang S. & Murphy T. H. Imaging the impact of cortical microcirculation on synaptic structure and sensory-evoked hemodynamic responses in vivo. PLoS Biol 5, e119 (2007). 17456007 10.1371/journal.pbio.0050119 PMC1854912

[b60] Drew P. J., Blinder P., Cauwenberghs G., Shih A. Y. & Kleinfeld D. Rapid determination of particle velocity from space-time images using the Radon transform. J Comput Neurosci 29, 5–11 (2010). 19459038 10.1007/s10827-009-0159-1 PMC4962871

[b61] Li Y. *et al.* Direct labeling and visualization of blood vessels with lipophilic carbocyanine dye DiI. Nat Protoc 3, 1703–1708 (2008). 18846097 10.1038/nprot.2008.172 PMC2811090

[b62] Sribnick E. A. *et al.* Postinjury estrogen treatment of chronic spinal cord injury improves locomotor function in rats. J Neurosci Res 88, 1738–1750 (2010). 20091771 10.1002/jnr.22337 PMC3127445

[b63] Basso D. M. *et al.* Basso Mouse Scale for locomotion detects differences in recovery after spinal cord injury in five common mouse strains. J Neurotraum 23, 635–659 (2006). 10.1089/neu.2006.23.635 16689667

